# Molecular classification of non-invasive breast lesions for personalised therapy and chemoprevention

**DOI:** 10.18632/oncotarget.6525

**Published:** 2015-12-09

**Authors:** Niamh Buckley, David Boyle, Darragh McArt, Gareth Irwin, D. Paul Harkin, Tong Lioe, Stephen McQuaid, Jacqueline A. James, Perry Maxwell, Peter Hamilton, Paul B. Mullan, Manuel Salto-Tellez

**Affiliations:** ^1^ Centre for Cancer Research and Cell Biology, Queen's University Belfast, Belfast, United Kingdom; ^2^ Department of Histopathology, Belfast City Hospital, Belfast, United Kingdom

**Keywords:** pre-invasive breast cancer, DCIS, personalised medicine, biomarker, molecular pathology, Pathology Section

## Abstract

Breast cancer screening has led to a dramatic increase in the detection of pre-invasive breast lesions. While mastectomy is almost guaranteed to treat the disease, more conservative approaches could be as effective if patients can be stratified based on risk of co-existing or recurrent invasive disease.

Here we use a range of biomarkers to interrogate and classify purely non-invasive lesions (PNL) and those with co-existing invasive breast cancer (CEIN). Apart from Ductal Carcinoma *in situ* (DCIS), relative homogeneity is observed. DCIS contained a greater spread of molecular subtypes. Interestingly, high expression of p-mTOR was observed in all PNL with lower expression in DCIS and invasive carcinoma while the opposite expression pattern was observed for TOP2A.

Comparing PNL with CEIN, we have identified p53 and Ki67 as predictors of CEIN with a combined PPV and NPV of 90.48% and 43.3% respectively. Furthermore, HER2 expression showed the best concordance between DCIS and its invasive counterpart.

We propose that these biomarkers can be used to improve the management of patients with pre-invasive breast lesions following further validation and clinical trials. p53 and Ki67 could be used to stratify patients into low and high-risk groups for co-existing disease. Knowledge of expression of more actionable targets such as HER2 or TOP2A can be used to design chemoprevention or neo-adjuvant strategies. Increased knowledge of the molecular profile of pre-invasive lesions can only serve to enhance our understanding of the disease and, in the era of personalised medicine, bring us closer to improving breast cancer care.

## INTRODUCTION

A variety of non-invasive lesions are encountered in breast tissue whether derived from resection specimens or core biopsy material. An increased understanding of the prognosis associated with the prototypical pre-invasive lesion ductal carcinoma *in situ* (DCIS) is required to both adequately manage and avoid overtreatment [1]. Predictive and prognostic biomarkers are required; not only for low-risk patients but also in high grade DCIS as its patient-specific propensity to progress to invasion is imprecisely represented by clinicopathologic characteristics and standard biomarkers [2].

Breast screening programmes have increased not only the frequency of *in situ* disease detection but also others such as columnar cell lesions (CCL). The proportion of UK screening detected cancers classified as *in situ* was 20% during 2013/2014 [3]. During the same period the proportion of biopsied lesions classified as benign was 9%. The detection of lesions, for example CCL, may be clinically important: whilst they are not in themselves aggressive they are frequently associated with particular invasive carcinoma types such as low grade infiltrating ductal and classic lobular carcinoma [4, 5]. Therefore how should these non-invasive lesions be managed when present in apparent isolation and how can they be further characterised?

While simple mastectomy will undoubtedly effectively treat *in situ* carcinoma and other non-invasive lesions, more conservative resection with or without radiotherapy may achieve similar success [6, 7]. In essence, the addition of radiotherapy or systemic therapy to surgical intervention is insurance against the minority of patients who will subsequently present with invasive cancer [8]. A recent retrospective study from Narod *et al*. analysed over 100,000 cases of DCIS with extensive follow-up data [9]. They found that aggressive treatment did not reduce the number of breast cancer associated deaths highlighting the need for novel treatment strategies. They also demonstrate that occurrence of invasive cancer after DCIS substantially increases the risk of cancer-related death. Given the fact that the characteristics of DCIS can predict those of subsequent invasive disease, more robust characterisation of DCIS can inform preventative strategies. Currently, no reliable diagnostic approach exists to identify lesions likely to progress or to become invasive. Therefore other parameters, not only to treat but also to help decide the risk of recurrence or progression are required.

We sought to determine the molecular characteristics of lesions associated with invasive carcinoma in comparison with lesions existing in a pure form (purely non-invasive lesions, or PNL in this manuscript) in terms of established biomarkers and potential druggable targets [10]. By measuring possible differences in expression, we sought to infer likely co-existence of invasive carcinoma (CEIN) in breast specimens thereby identifying markers of possible prognostic utility. We tested the hypothesis that alterations in biomarker expression would occur across lesions representative of a spectrum from morphologically normal, benign, *in situ* and invasive carcinoma.

## RESULTS

**Figure 1:**
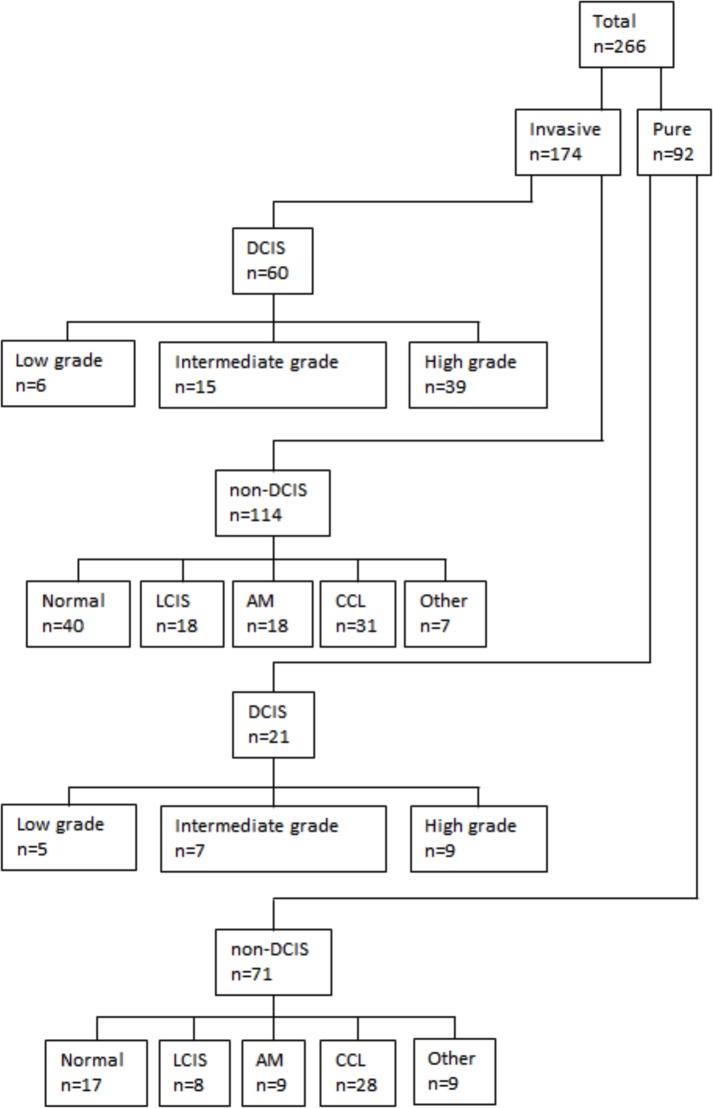
Consort diagram of study cohort

The consort diagram and derivation of cases is summarised in Figure 1. Patient clinicopathologic information for CEIN lesions is presented in Supplementary Table 2. Median and modal ages of patients with PNLs were 50 and 53 respectively (range 18-80 years). A total of 266 non-invasive lesions were available for analysis, following exclusion of cases missing blocks or if lesions cut out during sectioning and staining. The PNL group comprised 17 normal samples; 9 apocrine metaplastic lesions; 28 CCL; 8 LCIS; 21 DCIS; 9 others represented a range of infrequently encountered lesions. The CEIN group comprised 40 ‘normal’ samples (terminal ductulo-lobular units); 18 apocrine metaplastic lesions; 31 CCL; 18 LCIS; 60 DCIS; 7 others represented a range of infrequently encountered lesions (Supplementary Table 4). The following results pertain to normal, apocrine metaplasia (AM), CCL, LCIS, DCIS and invasive carcinoma.

### Expression of molecular subtypes according to lesion type

We assessed the frequency of molecular subtypes per lesion class. Fifty-seven examples of morphologically normal ductulo-lobular units were assessable of which 98% displayed luminal expression. The single non-luminal example had a triple negative phenotype and was from the CEIN group. Ninety-six percent of luminal examples had a luminal A phenotype. Another CEIN case was classified as LBHN due to high Ki67 expression. Two cases could not be refined beyond luminal classification. The 24 classifiable cases of AM were all of a triple negative phenotype.

Fifty-eight examples of CCL were assessable, all having a luminal phenotype. Ninety-two percent of these were of luminal A phenotype. The remaining examples were of LBHN phenotype and displayed morphologic features of FEA (flat epithelial atypia): namely, columnar cells with non-basally orientated rounded nuclei and mild atypia.

Of the twenty-six cases of LCIS, 80% were defined as luminal A and 20% as LBHN. All but one of these was CEIN related. One case could not be further defined. Eighty-four percent of the eighty examples of DCIS were of luminal phenotype. In this group of DCIS type lesions 39% were luminal A, 21% LBHN, 24% LBHP, 6% HE and 10% TN phenotype. DCIS from the CEIN group showed luminal phenotype in 81% of cases. The remaining cases contained all the TN phenotype tumours. In the PNL group, luminal phenotype was defined in 86% of cases.

### Morphologic and molecular phenotypic features of DCIS

Given that DCIS, compared to the other lesion types studied, has the greatest potential for progression to invasion, although the rate at which this occurs is variable, we explored the relationship between morphologic appearances and molecular phenotype. Fewer than half of luminal DCIS cases (45%) had associated comedonecrosis whereas a greater proportion (77%) of non-luminal cases showed this feature. When limited to PNL, a 33% of cases displayed comedonecrosis whereas 55% of CEIN lesions demonstrated this.

All but one of the non-luminal DCIS examples were high grade (92%), the other being classed as intermediate grade. Luminal DCIS was dominated by high grade examples (52%) but contained a greater proportion of intermediate (31%) and low grades (16%). Within the PNL group, 43% of DCIS was high grade, 33% intermediate grade and 24% low grade. This is significantly different to CEIN DCIS where a greater proportion was high grade (65%) with fewer cases showing intermediate (25%) and low grade (10%) morphology (*p*-value 0.0034).

### Expression of novel biomarkers

**Figure 2:**
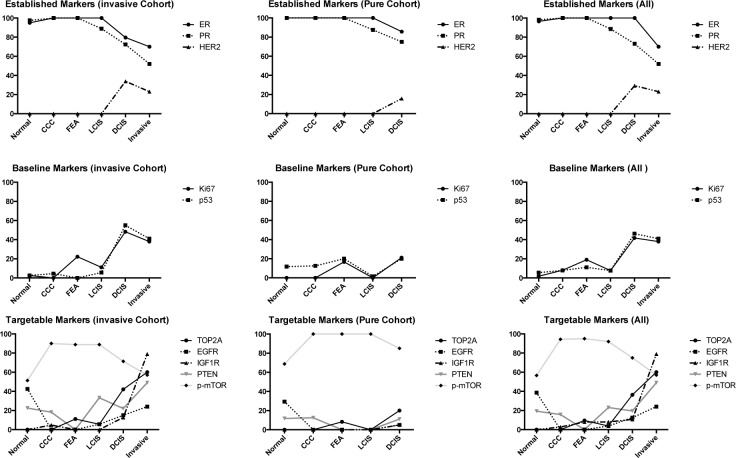
Trends of expression of biomarkers within different lesion subtypes

Having described the non-invasive case cohort in terms of molecular phenotype we then wished to characterise these cases in terms of more novel biomarker targets both singly (Figure 2) and within molecular subclasses (Supplementary Tables 5 and 6). TOP2A expression occurred in 36% of DCIS with expression occurring seldom or not at all in other lesions. The majority of cases expressing TOP2A were high grade (none were low grade) and usually associated with invasive carcinoma.

EGFR expression was frequent in normal (39%) and apocrine metaplasia (50%), absent in CCL and present in LCIS (4%) and DCIS (13%). Expression was faint in benign lesions while DCIS and invasive carcinoma occasionally showed more intense expression. The majority of DCIS cases expressing EGFR were CEIN related (9 of the 10 positive cases had associated invasive carcinoma), mostly high grade or intermediate grade, and almost all with comedonecrosis. EGFR expression was relatively more frequent in non-luminal examples of DCIS. All of the normal examples with EGFR expression were LA phenotype.

Loss of IGF1R occurred most often in apocrine metaplasia (40%) followed by DCIS (11%). In the small number of DCIS cases with loss, the majority were high grade (88% or 100% when intermediate was included) and related to CEIN. Additionally, 75% of cases showing loss also demonstrated comedonecrosis. The few DCIS cases with complete loss were LBHP or TN while all AM examples were TN.

**Figure 3:**
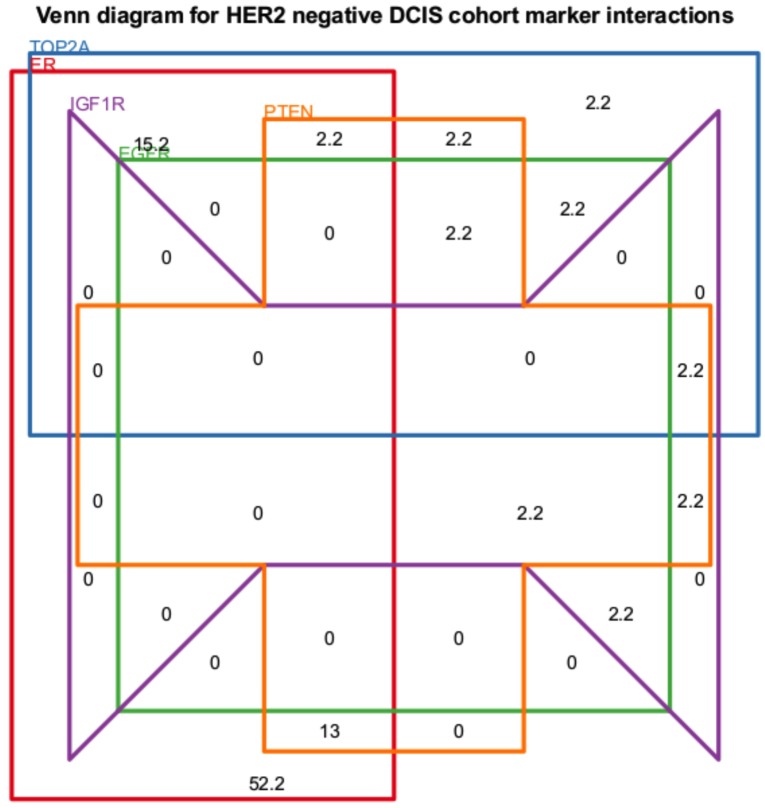
Venn diagram displaying the biomarker expression overlaps for HER2 negative DCIS cases All values are percentages of cases. PTEN and IGF1R refer to expression loss. All other biomarkers refer to over-expression.

PTEN assessment was difficult in a number of cases, posing similar challenges in interpretation reported by other groups due to variations in intensity and the presence of both cytoplasmic and nuclear expression [11, 12]. PTEN loss occurred most frequently in LCIS (23%) but at similar rates in normal (19%), CCL (16%) and DCIS (20%): in all of these examples loss of PTEN was most frequent with CEIN lesions (Figure 3). A single PNL apocrine metaplastic focus demonstrated PTEN loss. In the DCIS PTEN loss group, 87% were high grade or intermediate grade. Comedonecrosis occurred in 47% of PTEN loss cases and 53% of PTEN retention cases. In DCIS, PTEN loss occurred in almost all molecular phenotypes.

Aside from apocrine metaplasia, the majority of lesions of each type expressed p-mTOR particularly in the PNL group. mTOR over-expression occurred in CCL (94%), FEA (95%) and LCIS (92%). 75% of DCIS expressed p-mTOR. Just over half of positive cases (53%) had associated comedonecrosis especially from the CEIN group. Thirty-nine percent of p-mTOR negative cases had comedonecrosis. p-mTOR over-expression occurred in all DCIS molecular phenotypes.

### Differences in biomarker expression between matched DCIS and invasive carcinoma

The level of agreement between the molecular phenotype of DCIS and invasive carcinoma in specific cases was explored. Overall, by Cohen's kappa coefficient, the degree of agreement was moderate (κ = 0.561; 95% confidence interval: 0.332 to 0.791). While the majority of cases showed broadly concordant luminal status molecular phenotypic expression between *in situ* and invasive disease, in ten cases were discordant. This increased to at least 21 when precise molecular phenotype was defined. Discordant cases are summarised in Supplementary Table 3.

The level of agreement, or lack of change, between individual biomarkers assessed in DCIS and in their invasive counterparts is shown in Table 1. The best concordance was demonstrated by HER2. Moderate concordance was found with TOP2A, EGFR, p53, ER and PR. The remainder showed fair to poor concordance. Apart from HER2, as the remaining biomarkers showed less concordance between DCIS and their invasive counterparts, we calculated the overlap of biomarker expressions in HER2 negative cases (Figure 3). The most often expressed biomarkers in the remaining cases were ER (82.6%) and TOP2A (28.4%): 17.4% of cases co-expressed both biomarkers. This also demonstrates the complexity of biomarker expression within DCIS, which underlies the lack of clear therapeutic avenues.

**Table 1:** Concordance between biomarker scores in DCIS and related invasive lesions

Biomarker	Kappa	Interpretation
ER	0.581	moderate
PR	0.418	moderate
HER2	0.839	very good
Ki67	0.298	fair
p53 NE *vs* EP/EN	0.336	fair
p53 EN *vs* NE *vs* EP	0.457*	moderate
TOP2A	0.423	moderate
EGFR	0.522	moderate
IGF1R	0.387	fair
PTEN	0.339	fair
p-mTOR	0.153	poor

Kappa Coefficient interpretation index: 0-0.20 = Poor, 0.21-0.40 = Fair, 0.41-0.60= Moderate, 0.61-0.80 = Substantial, 0.81-1 = Almost Perfect.

* weighted kappa = 0.540.

In terms of biomarker relation to sample type, only p53 expression showed a significant relation (*p* = 0.0091) with sample type, tending to be positive when DCIS was associated with a co-existent invasive carcinoma (Table 2). Similarly, Ki67 tended (*p* = 0.060) to indicate high proliferation when DCIS was associated with invasive carcinoma compared to DCIS existing purely alone. The positive predictive value (PPV) and negative predictive values (NPV) for p53 alone in this situation were 89.19% and 37.21% respectively. Ki67 alone produced a PPV of 87.85% and NPV of 32.58%. However, assessing agreement of both biomarkers produced the best results with a PPV of 90.48% and NPV of 43.33%. Sensitivity and specificity were 53% and 87% respectively. Performing the analysis when assessing both or either biomarker positivity did not improve predictive values further; however, sensitivity and specificity were both 72% in this instance. Calculations of the relationship between sample type and luminal *versus* non-luminal status, DCIS grade and presence of comedonecrosis were not significant (data not shown).

**Table 2:** Fisher's exact test contingency table analysis of biomarker expression between PNL and CEIN

	Normal	AM	CCC/FEA	LCIS	DCIS
**ER**	NS	NS	NS	NS	NS
**PR**	NS	NS	NS	NS	NS
**HER2**	NS	NS	NS	NS	NS
**Ki67**	NS	NS	NS	NS	0.06*
**P53**	NS	NS	NS	NS	0.0091**
**TOP2A**	NS	NS	NS	NS	0.1063
**EGFR**	NS	NS	NS	NS	NS
**IGF1R**	NS	NS	NS	NS	NS
**PTEN**	NS	NS	NS	NS	NS
**p-mTOR**	NS	NS	NS	NS	NS

## DISCUSSION

In this study, we investigated the molecular characteristics of lesions associated with invasive carcinoma (CEIN) *versus* purely non-invasive lesions (PNL) with the aim of identifying potential prognostic biomarkers as well as potential targets for chemoprevention. Apart from DCIS, pre-invasive lesions are a relatively homogenous group, dominated by a luminal phenotype. As may be expected from higher association with progression, DCIS is a much more heterogeneous group with a spectrum of molecular classifications and biomarker expression. This is in keeping with a recent publication, using multiple platforms to present a comprehensive molecular portrait, demonstrating multiple molecular subgroups within DCIS [13]. Of note, p-mTOR expression was high in most benign and pre-neoplastic lesions with reduced expression in DCIS and invasive carcinoma. TOP2A expression was associated with DCIS, usually high grade and associated with invasive carcinoma. We demonstrate that p53 and Ki67 expression may predict the association of DCIS with invasive carcinoma and that HER2 shows the best concordance between *in situ* and invasive lesions.

The importance of better describing risk in DCIS relates to it not being the inevitable harbinger of invasive carcinoma, not only because a spectrum of lesion aggressiveness exists but as natural lesion evolution may span several decades. Consequently, most consider DCIS a non-obligate precursor to invasive breast carcinoma. However, at least one study has shown that untreated DCIS is at increased risk of progression regardless of grade of *in situ* disease although lower grade lesions may progress more slowly [14]. In our study we found no relationship between DCIS grade or comedonecrosis and association with invasion.

The emphasis in breast cancer management is increasingly towards earlier invasive lesions and pre-invasive stages of disease. Early detection provides opportunities but also new challenges in correctly determining the significance of lesions to avoid overtreatment [15]. Preventative approaches are preferable to managing invasive carcinoma, not only because of reduced risk of distant spread but also the avoidance or reduction of treatment resistance and possibility for less drastic intervention. Strategies for prevention are facilitated by increasingly sensitive lesion detection and availability of screening. This might also facilitate active surveillance of specific non-invasive lesions at low risk of progression and use of p53 and Ki67, as described here, may help realise these aims [16].

In cases where only DCIS is detected, the inevitable question is can the use of biomarkers influence patient care? Biomarkers such as p53 and Ki67 would be used to stratify based on likelihood of presence of an undetected co-existing invasive carcinoma. It would be inferred that women at low risk of CEIN could be offered more conservative surgery with or without radiotherapy based on additional clinical parameters. On the other hand, high-risk patients would be offered full mastectomy and/or radiotherapy and/or additional systemic treatment as determined by biomarker status and additional clinical information. In addition, as IHC-based biomarker assessment can be carried out on core biopsy material, this mean that these parameters can be taken into account at the time of diagnosis rather than waiting for the excision biopsy when surgery has already been performed.

Other studies have examined the utility of biomarkers in predicting subsequent invasive carcinoma. Kerlikowske et al studied the characteristics of patients with DCIS who later developed invasive carcinoma [17]. In the 170 patients with progression, a combination of Ki67 positivity with positivity for p16 and COX2 in the initial *in situ* lesion was significantly associated with subsequent invasion. Similar findings showing increased risk of subsequent *in situ*, but not invasive recurrence were reported by Rakovitch et al. using a combination of HER2 and Ki67 positivity [18]. Ki67 positivity in our study was associated but not significantly (*p* = 0.06) with greater likelihood of presence of invasion. A small study addressing rare cases of distant metastases following an initial diagnosis and treatment for DCIS may allude to occult invasive foci or particularly aggressive disease [19]. In this series 74% of patients with distant recurrences had DCIS with necrosis while almost two thirds were ER negative hinting towards an aggressive non-luminal phenotype.

Of the standard-of-care predictive biomarkers and additional potential actionable targets, HER2 showed the best concordance between *in situ* and invasive carcinoma in individual cases. This implies that DCIS surrounding invasive carcinoma is most similar to its HER2 invasive counterpart. Consequently, if a patient is identified as being at higher risk of CEIN, and the DCIS is HER2 positive, use of HER2 targeted therapies would be of benefit in not only targeting the DCIS but also any associated undetected invasive carcinoma. Conversely, treatment of HER2 positive invasive carcinoma will also be likely to target co-existing DCIS. When HER2 positive DCIS was excluded the most often expressed biomarkers in the remaining cases were ER (82.6%) and TOP2A (28.4%) with 17.4% of cases co-expressing both. TOP2A was associated with higher grade DCIS and a tendency for concurrent invasive disease similar to other reported findings [20, 21]. Hormone receptor and anthracycline based therapies may be indicated in these cases [22].

Few trials exist to test the hypothesis that non-surgical intervention may be optimal in at least a subgroup of patients with DCIS. Most published work evaluates protocols to predict the risk of tumour recurrence (*in situ* and invasive) following excision [23]. However, the cancer and leukaemia group B (CALGB) 40903 study (NCT01439711) aims to determine what subsets of ER positive DCIS might be amenable to neoadjuvant endocrine therapy with the proviso that all patients will eventually have surgical excision. Such intervention may cause complete lesion regression or reduce lesional size to an extent where more localised resection is possible. A randomised phase I/II trial (NCT00555152), yet to report, is assessing the effects of neoadjuvant lapatinib (a tyrosine kinase inhibitor causing dual inhibition of HER2 and EGFR) in DCIS by measuring changes in Ki67 index at the time of surgical excision. DeCensi et al. previously reported on the effects of lapatinib in HER2 positive invasive carcinoma and surrounding lesions, including DCIS, in 60 women. They found non-significant (*p* = 0.067) reductions in the mean Ki67 index in patients treated neoadjuvantly with lapatinib [24]. The neoadjuvant effects of trastuzumab on DCIS have been assessed by Kuerer et al. [25]. From an initial 69 eligible patients, 24 had in situ lesions with HER2 overexpression and 12 of these received the drug. The single pre-operative dose did not produce any significant pathologic, anti-proliferative or anti-apoptotic effects as assessed on resection specimens. Notably, 5 patients showed evidence of invasive disease in resection specimens. Another trial is expected to report on the benefit of trastuzumab with radiotherapy given to patients with HER2 positive DCIS following lumpectomy [26]. This is an important option in hormone receptor negative DCIS and estimates suggest that approximately 40 - 50% of pure DCIS may be HER2 positive [27, 28]. Our findings would suggest that targeted biomarker informed therapies could be used in the neo-adjuvant setting to potentially negate the need for surgery completely. Furthermore, given the increasing emphasis on knowledge of tumour heterogeneity and its potential influence on multiple aspects of tumour biology including progression, spread and treatment response, molecular characterisation of co-existing DCIS may aid in clinical management decisions in the future.

In summary, the benign and early neoplastic lesions described herein display homogeneity in their molecular profiles. The greatest diversity of biomarker expression occurs at the *in situ* carcinoma phase of non-invasive lesions. Defining the molecular profile of *in situ* disease may help in deciphering the likelihood of related invasive disease, the risk or recurrence and hence inform management decisions. Enhancing our understanding of both similarities and differences between non-invasive and invasive lesions may aid in predicting the response to treatment.

## MATERIALS AND METHODS

### Case identification

Ethical approval was obtained and tissue acquired for this research through the Northern Ireland Biobank (NIB ref: 12-00017). Patients with PNLs had no previous breast cancer and no cancer developed since diagnosed, while patients with invasive carcinoma all underwent surgery and adjuvant chemotherapy. For PNLs, specimens were identified through our hospital laboratory database during the 2008 to 2011 period. Non-invasive diagnostic terms were compiled into a search limited to complete mastectomies and wide local excisions: core biopsies were excluded. The slides from all available cases were reviewed to confirm diagnostic coding and the most representative blocks selected for biomarker analysis. Only lesions of sufficient size or extent unlikely to cut out during sectioning were selected for further analysis. CEIN lesions were derived from a previous study originating from the same institutional archival material and following slide review, representative blocks were selected for further analysis [29, 30]. All of the material was reviewed by 2 breast cancer pathologists (DPB and TFL).

### Lesion diagnoses

Classification and grading of DCIS was performed according to UK national guidance [31]. Grading was defined as low, intermediate or high. The presence of comedonecrosis was recorded. Columnar cell lesions were diagnosed and categorised with the nomenclature suggested by Schnitt et al. [32, 33]. All other lesions were assessed according to standard diagnostic criteria (Supplementary Table 1).

### Immunohistochemical analyses

Immunohistochemical analysis for ER, PR, HER2, Ki67, p53, TOP2A, EGFR, IGF1R, PTEN and p-mTOR was performed using automated immunostainers. The specific clones, internally optimised antibody conditions (dilution and pre-treatment) and automated platform used are presented in Supplementary Table 1. The expression of each biomarker was assessed in the tumour or normal epithelial component of each region of interest under investigation. The majority of the biomarkers were subjectively scored by semi-quantitative analysis according to standards previously set out in the literature and discussed in a recent review [10]. Briefly, twelve whole tissue sections for IHC were cut at 4 microns on a rotary microtome, dried at 37°C overnight and then used for IHC assays on Ventana DISCOVERY^®^ XT or Leica BOND-MAX™ automated immunostainers. Analyses were limited to lesions with at least 5 examples. These biomarkers were used because they are a) key for the classification of breast cancer; and/or b) are potential companion diagnostics for drugs tested in early phase clinical trials in invasive breast cancer.

Molecular subtype assignation was performed using a cohort of markers (ER, PR, HER2, Ki67) in accordance with recent recommendations [34]. The 5 molecular subtypes were defined as luminal A (ER^+^, PgR^+^, HER2^−^ Ki67 low), luminal B HER2 negative (ER^+^, HER2^−^ and at least one of Ki67 high or PgR), luminal B HER2 positive (ER^+^, HER2^+^), HER2 enriched (HER2^+^, ER^−^ and PgR^−^) and triple-negative (ER^−^, PgR^−^, HER2^−^). Cases were also dichotomised, where possible, as luminal or non-luminal when a full cohort of biomarkers was unavailable. A number of representative images are shown in Supplementary Figure1.

### Statistical analyses

Statistical analyses were performed using GraphPad PRISM and R (https://CRAN.R-project.org) with Venn diagrams constructed using the R package Vennerable (http://R-Forge.R-project.org/projects/vennerable). Measures of agreement for biomarker interpretation between DCIS and invasive carcinoma were expressed by calculating kappa values [35]. Quantification of the relationship between biomarkers and their expression in particular sample types was performed using contingency tables and analysis by Fisher's exact test or chi-square test as appropriate. *P*-values were not multiple hypothesis corrected. Following this, for significant results, biomarker positive and negative predictive values were calculated.

## SUPPLEMENTARY MATERIAL TABLES AND FIGURE


