# Supplementary material for: Molecular classification of non-invasive breast lesions for personalised therapy and chemoprevention
Source: Oncotarget. 2015 Dec 9;6(41):43244–54. doi: 10.18632/oncotarget.6525 (PMC4791229; doi:10.18632/oncotarget.6525)
Supplement: Supplementary file 1 [file oncotarget-06-43244-s001.pdf]

Molecular classification of non-invasive breast lesions for personalised therapy and chemoprevention

Supplementary Material

Supp Table 1 – Antibody conditions and thresholds for over or under expression.

Antibody	Clone	Dilution/Pre-treatment	Company	Automated platform	Threshold
ER	6F11	1:200/ER2 20mins	Leica	Leica BOND-MAX	Nuclear expression on at least 1% cells (34)
PR	636	1:150/ER2 20mins	Dako	Leica BOND-MAX	Nuclear expression on at least 1% cells
HER2	CB11	Oracle (pre-set)	Leica	Leica BOND-MAX	Membrane expression. Only 3+ assessed as positive
p53	DO-7	1:100/ER1 30mins	Dako	Leica BOND-MAX	Nuclear expression. Extremes of positive and negative considered aberrant (35)
Ki67	MM1	1:200/ER2 30mins	Leica	Leica BOND-MAX	nuclear expression in >5% of cells
TOP2a	JS5B4	Pre-set/CC1 mild	Ventana	Ventana DISCOVERY XT	nuclear expression in >5% of cells
EGFR	3C6	Pre-set/Protease 12mins	Ventana	Ventana DISCOVERY XT	Any degree of membrane bound expression
IGF1R	G11	Pre-set/CC1 mild	Ventana	Ventana DISCOVERY XT	membrane expression in <5% of cells = loss
PTEN	6H2.1	1:1600/ER2 20mins	Dako	Leica BOND-MAX	cytoplasmic expression on <1% cells =loss
p-mTOR	49F9	1:100/ER2 20mins	Cell Signalling	Leica BOND-MAX	cytoplasmic expression on >5% of cells

Supp Table 2. Patient clinicopathologic information for those with an invasive carcinoma component. (Abbreviations: NPI, Nottingham Prognostic Index; LVI, lymphovascular invasion; ER, oestrogen receptor; LA, luminal A; LBHN, luminal B HER2 negative; LBHP, luminal B HER2 positive; HE, HER2 enriched; TN, triple negative; OS (60), overall survival at 60 months)

ALL LESIONS		CEIN, n (%)
Age	Median	48
pN stage	0	27 (35)
	1	24 (31)
	2	10 (13)
	3	10 (13)
pT stage	1	18 (23)
	2	44 (57)
	3	7 (9)
	4	2 (3)
NPI	>3.4 but <=5.4	50 (65)
	>5.4	21 (27)
LVI	Present	41 (53)
	Absent	30 (39)
invasive type	Ductal	52 (68)
	Lobular	8 (10)
	Mixed	11 (14)
tumour grade	1	3 (4)
	2	36 (47)
	3	32 (42)
ER	Positive	50 (65)
	negative	21 (27)
HER2	positive	16 (21)
	negative	55 (71)
Tumour classification	LA	25 (32)
	LBHN	17 (22)
	LBHP	8 (10)
	HE	8 (10)
	TN	13 (17)
	Luminal	50 (65)
	Non-luminal	21 (27)
OS (60)	yes	8 (10)
	no	63 (82)

Supp Table 3 Molecular summary of discordant cases

**Molecular classification**		**Luminal phenotype change**
**DCIS**	**Invasive**	
LA	LBHN	No
LA	LBHN	No
LA	LBHN	No
LA	LBHN	No
LA	LBHN	No
LBHN	HE	Yes
LBHN	LA	No
LBHN	LA	No
LBHN	LA	No
LBHN	LBHP	No
LBHN	TN	Yes
LBHN	TN	Yes
LBHP	HE	Yes
LBHP	HE	Yes
LBHP	HE	Yes
LBHP	HE	Yes
LBHP	LA	No
LBHP	LBHN	No
NULL (luminal)	HE	Yes
NULL (nonluminal)	HE	No
NULL (luminal)	LA	No
NULL (luminal)	LA	No
NULL (luminal)	LBHN	No
NULL (NULL)	LBHN	unknown
NULL (luminal)	TN	Yes
NULL (NULL)	TN	unknown
TN	LA	Yes

Supp Table 4: Pathological classification of pre-invasive lesions.

	Total	Pure	Invasive
**DCIS**	81	21	60
**LCIS**	26	8	18
**CCL**	59	28	31
**AM**	27	9	18
**Normal**	57	17	40

	Pure	Invasive	Totals
**ADH**	2	1	3
**ALH**	1	0	1
**AM**	9	18	27
**Atypical apocrine metaplasia**	0	1	1
**CCC**	16	22	38
**FEA**	12	9	21
**DCIS**	21	60	81
**Encysted papillary carcinoma**	1	0	1
**Fibroadenoma**	0	1	1
**HUT**	3	2	5
**Papilloma**	2	1	3
**LCIS**	8	18	26
**MGA**	0	1	1
**Normal**	17	40	57
**Totals**	92	174	

Supp Table 5: Quantification of biomarker expression within pathological subgroups.

Sample Type	Lesion	ER (%)	PR(%)	HER2 (%)	Ki67 (%)	p53 (%)	TOP2A (%)	EGFR (%)	IGF1R (%)	PTEN (%)	p-mTOR (%)
CEIN	normal	38/40 (95)	39/40 (97.5)	0/40 (0)	1/40 (2.5)	1/38 (2.6)	0/37 (0)	17/40 (42.5)	0/38 (0)	9/40 (22.5)	19/37 (51.4)
	apocrine metaplasia	0/16 (0)	0/18 (0)	0/18 (0)	0/17 (0)	1/15 (6.7)	0/17 (0)	8/17 (47.1)	7/§6 (43.8)	0/17 (0)	3/17 (17.6)
	CCC	20/20 (100)	21/21 (100)	0/21 (0)	0/22 (0)	1/22 (4.5)	0/22 (0)	0/22 (0)	1/21 (4.8)	4/22 (18.2)	20/22 (90.9)
	FEA	9/9 (100)	9/9/(100)	0/9 (0)	2/9 (22.2)	0/8 (0)	1/9 (11.1)	0/9 (0)	0/9 (0)	0/9 (0)	8/9 (88.9)
	LCIS	18/18 (100)	16/18 (88.9)	0/18 (0)	2/18 (11.1)	1/18 (5.6)	1/18 (5.6)	1/18 (5.6)	0/18 (0)	6/18 (33.3)	16/18 (88.9)
	DCIS	47/59 (79.6)	42/58 (72.4)	19/56 (33.9)	29/60 (48.3)	33/60 (55)	24/57 (42.1)	9/60 (15)	7/55 (12.7)	13/59 (22)	40/56 (71.4)
	Invasive	50/71 (70)	37/71 (52)	16/71 (23)	26/29 (38)	29/71 (41)	41/68 (60)	17/71 (24)	55/70 (79)	33/68 (49)	40/70 (57)
PNL	normal	17/17 (100)	17/17 (100)	0/17 (0)	0/16 (0)	2/17 (11.8)	0/17 (0)	5/17 (29.4)	0/17 (0)	2/17 (11.8)	11/16 (68.75)
	apocrine metaplasia	0/9 (0)	0/9 (0)	0/8 (0)	0/9 (0)	2/9 (22.2)	0/9 (0)	5/9 (55.6)	3/9 (33.3)	2/9 (22.2)	4/8 (50)
	CCC	15/15 (100)	16/16 (100)	0/15 (0)	0/15 (0)	2/16 (12.5)	0/15 (0)	0/15 (0)	0/15 (0)	2/16 (12.5)	14/14 (100)
	FEA	12/12 (100)	11/11 (100)	0/11 (0)	2/12 (16.7)	2/10 (20)	1/12 (8.3)	0/12 (0)	0/12 (0)	2/10 (20)	11/11 (100)
	LCIS	7/7 (100)	7/8 (87.5)	0/8 (0)	0/8 (0)	1/8 (12.5)	0/8 (0)	0/8 (0)	0/7 (0)	1/8 (12.5)	7/7 (100)
	DCIS	18/21 (85.7)	15/20 (75)	3/19 (15.8)	4/19 (21.1)	4/20 (20)	4/20 (20)	1/20 (5)	1/20 (5)	4/20 (20)	17/20 (85)
all	normal	55/57 (96.5)	56/57 (98.2)	0/57 (0)	1/56 (1.8)	3/55 (5.5)	0/54 (0)	22/57 (38.6)	0/55 (0)	11/57 (19.3)	30/53 (56.6)
	apocrine metaplasia	0/25 (0)	0/27 (0)	0/26 (0)	0/26 (0)	3/24) 12.5)	0/26 (0)	13/26 (50)	10/25 (40)	1/26 (3.8)	7/25 (28)
	CCC	35/35 (100)	37/37 (100)	0/36 (0)	0/37 (0)	3/38 (7.9)	0/37 (0)	0/37 (0)	1/36 (2.8)	6/38 (15.8)	34/36 (94.4)
	FEA	21/21 (100)	20/20 (100)	0/20 (0)	4/21 (19)	2/18 (11.1)	2/21 (9.5)	0/21 (0)	0/21 (0)	0/21 (0)	19/20 (95)
	LCIS	25/25 (100)	23/26 (88.5)	0/26 (0)	2/26 (7.7)	2/26 (7.7)	1/26 (3.8)	1/26 (3.8)	0/25 (0)	6/26 (23.1)	23/25 (92)
	DCIS	55/80 (81.25)	57/78(73.1)	22/75 (29.3)	33/79 (41.8)	37/80 (46.3)	28/77 (36.4)	10/80 (12.5)	8/75 (10.7)	15/77 (19.5)	57/76 (75)
	Invasive	50/71	37/71 (52)	16/71	26/29	29/71	41/68	17/71	55/70	33/68	40/70

		(70)		(23)	(38)	(41)	(60)	(24)	(79)	(49)	(57)

Supp Table 6: Quantification of biomarker expression within molecular subgroups.

DCIS (n=81)	LA	LBHN	LBHP	HE	TN	*Unclassified*	Luminal	Non-luminal	*Unclassified*
ER	27 / 27 (100)	15 / 15 (100)	17 / 17 (100)	0 / 4 (0)	0 / 7 (0)	*6 / 10 (60)*	65 / 65 (100)	0 / 13 (0)	*0 / 2 (0)*
PR	27 / 27 (100)	12 / 15 (80)	12 / 16 (75)	0 / 4 (0)	0 / 7 (0)	*6 / 8 (75)*	55 / 62 (89)	0 / 13 (0)	*2 / 2 (100)*
HER2	0 / 27 (0)	0 / 15 (0)	17 / 17 (100)	4 / 4 (100)	0 / 7 (0)	*1 / 4 (25)*	17 / 60 (28)	4 / 11 (36)	*1 / 3 (33)*
TOP2a	3 / 27 (11)	5 / 15 (33)	10 / 15 (67)	3 / 4 (75)	4 / 7 (57)	*3 / 9 (33)*	20 / 61 (33)	7 / 13 (54)	*1 / 3 (33)*
EGFR	0 / 27 (0)	0 / 15 (0)	2 / 17 (12)	2 / 4 (50)	4 / 7 (57)	*2 / 10 (20)*	3 / 64 (5)	6 / 13 (46)	*1 / 3 (33)*
IGF1R	0 / 25 (0)	0 / 15 (0)	3 / 16 (19)	0 / 3 (0)	4 / 7 (57)	*1 / 9 (11)*	3 / 61 (5)	5 / 12 (42)	*0 / 2 (0)*
PTEN	4 / 25 (16)	4 / 15 (27)	1 / 17 (6)	0 / 3 (0)	4 / 7 (57)	*2 / 10 (20)*	10 / 62 (16)	4 / 12 (33)	*1 / 3 (33)*
p53	4 / 26 (15)	5 / 15 (33)	9 / 17 (53)	3 / 4 (75)	7 / 7 (100)	*9 / 11 (82)*	23 / 64 (36)	11 / 13 (85)	*3 / 3 (100)*
p-mTOR	20 / 26 (77)	9 / 14 (64)	15 / 17 (88)	3 / 3 (100)	2 / 6 (33)	*8 / 9 (89)*	48 / 62 (77)	6 / 10 (60)	*3 / 3 (100)*
Ki67	0 / 27 (0)	13 / 15 (87)	10 / 17 (59)	3 / 3 (100)	4 / 7 (57)	*3 / 10 (30)*	23 / 64 (36)	8 / 12 (67)	*2 / 3 (67)*
LCIS (n=26)	LA	LBHN	LBHP	HE	TN	*Unclassified*	Luminal	Non-luminal	*Unclassified*
ER	20 / 20 (100)	5 / 5 (100)	NA	NA	NA	*NA*	25 / 25 (100)	NA	*NA*
PR	20 / 20 (100)	2 / 5 (40)	NA	NA	NA	*1 / 1 (100)*	22 / 25 (88)	NA	*1 / 1 (100)*
HER2	0 / 20 (0)	0 / 5 (0)	NA	NA	NA	*0 / 1 (0)*	0 / 25 (0)	NA	*0 / 1 (0)*
TOP2a	1 / 20 (5)	0 / 5 (0)	NA	NA	NA	*0 / 1 (0)*	1 / 25 (4)	NA	*0 / 1 (0)*
EGFR	1 / 20 (5)	0 / 5 (0)	NA	NA	NA	*0 / 1 (0)*	1 / 25 (4)	NA	*0 / 1 (0)*
IGF1R	0 / 20 (0)	0 / 4 (0)	NA	NA	NA	*0 / 1 (0)*	0 / 24 (0)	NA	*0 / 1 (0)*
PTEN	4 / 20 (20)	2 / 5 (40)	NA	NA	NA	*0 / 1 (0)*	6 / 25 (24)	NA	*0 / 1 (0)*
p53	1 / 20 (5)	1 / 5 (20)	NA	NA	NA	*0 / 1 (0)*	2 / 25 (8)	NA	*0 / 1 (0)*
p-mTOR	20 / 20 (100)	3 / 5 (60)	NA	NA	NA	*NA*	23 / 25 (92)	NA	*NA*
Ki67	0 / 20 (0)	2 / 5 (40)	NA	NA	NA	*0 / 1 (0)*	2 / 25 (8)	NA	*0 / 1 (0)*
CCL (n=59)	LA	LBHN	LBHP	HE	TN	*Unclassified*	Luminal	Non-luminal	*Unclassified*

ER	49 / 49 (100)	4 / 4 (100)	NA	NA	NA	*3 / 3 (100)*	56 / 56 (100)	NA	*NA*
PR	49 / 49 (100)	4 / 4 (100)	NA	NA	NA	*4 / 4 (100)*	55 / 55 (100)	NA	*2 / 2 (100)*
HER2	0 / 49 (0)	0 / 4 (0)	NA	NA	NA	*0 / 3 (0)*	0 / 54 (0)	NA	*0 / 2 (0)*
TOP2a	0 / 48 (0)	1 / 4 (25)	NA	NA	NA	*1 / 6 (17)*	2 / 55 (4)	NA	*0 / 3 (0)*
EGFR	0 / 48 (0)	0 / 4 (0)	NA	NA	NA	*0 / 6 (0)*	0 / 55 (0)	NA	*0 / 3 (0)*
IGF1R	0 / 47 (0)	0 / 4 (0)	NA	NA	NA	*1 / 6 (17)*	0 / 54 (0)	NA	*1 / 3 (33)*
PTEN	6 / 49 (12)	0 / 4 (0)	NA	NA	NA	*0 / 6 (0)*	6 / 56 (11)	NA	*0 / 3 (0)*
p53	2 / 48 (4)	1 / 3 (33)	NA	NA	NA	*2 / 5 (40)*	5 / 53 (9)	NA	*0 / 3 (0)*
p-mTOR	47 / 49 (96)	3 / 4 (75)	NA	NA	NA	*3 / 3 (100)*	51 / 54 (94)	NA	*2 / 2 (100)*
Ki67	0 / 49 (0)	4 / 4 (100)	NA	NA	NA	*0 / 5 (0)*	4 / 56 (7)	NA	*0 / 2 (0)*
AM (n=27)	LA	LBHN	LBHP	HE	TN	*Unclassified*	Luminal	Non-luminal	*Unclassified*
ER	NA	NA	NA	NA	0 / 24 (0)	*0 / 1 (0)*	NA	0 / 25 (0)	*NA*
PR	NA	NA	NA	NA	0 / 24 (0)	*0 / 3 (0)*	NA	0 / 25 (0)	*0 / 2 (0)*
HER2	NA	NA	NA	NA	0 / 23 (0)	*0 / 2 (0)*	NA	0 / 23 (0)	*0 / 2 (0)*
TOP2a	NA	NA	NA	NA	0 / 24 (0)	*0 / 2 (0)*	NA	0 / 25 (0)	*0 / 1 (0)*
EGFR	NA	NA	NA	NA	12 / 23 (52)	*1 / 3 (33)*	NA	13 / 24 (54)	*0 / 2 (0)*
IGF1R	NA	NA	NA	NA	9 / 23 (39)	*1 / 2 (50)*	NA	9 / 24 (38)	*1 / 1 (100)*
PTEN	NA	NA	NA	NA	1 / 24 (4)	*0 / 2 (0)*	NA	1 / 25 (4)	*0 / 1 (0)*
p53	NA	NA	NA	NA	2 / 22 (9)	*1 / 2 (50)*	NA	2 / 23 (9)	*1 / 1 (100)*
p-mTOR	NA	NA	NA	NA	6 / 23 (26)	*1 / 2 (50)*	NA	7 / 24 (29)	*0 / 1 (0)*
Ki67	NA	NA	NA	NA	0 / 24 (0)	*0 / 2 (0)*	NA	0 / 25 (0)	*0 / 1 (0)*
Normal (n=57)	LA	LBHN	LBHP	HE	TN	*Unclassified*	Luminal	Non-luminal	*Unclassified*
ER	54 / 54 (100)	1 / 1 (100)	NA	NA	0 / 1 (0)	*0 / 1 (0)*	55 / 55 (100)	0 / 1 (0)	*0 / 1 (0)*
PR	54 / 54 (100)	1 / 1 (100)	NA	NA	0 / 1 (0)	*1 / 1 (100)*	55 / 55 (100)	0 / 1 (0)	*1 / 1 (100)*
HER2	0 / 54 (0)	0 / 1 (0)	NA	NA	0 / 1 (0)	*0 / 1 (0)*	0 / 55 (0)	0 / 1 (0)	*0 / 1 (0)*
TOP2a	0 / 51 (0)	0 / 1 (0)	NA	NA	0 / 1 (0)	*0 / 1 (0)*	0 / 52 (0)	0 / 1 (0)	*0 / 1 (0)*
EGFR	22 / 54 (41)	0 / 1 (0)	NA	NA	0 / 1 (0)	*0 / 1 (0)*	22 / 55 (40)	0 / 1 (0)	*0 / 1 (0)*
IGF1R	0 / 52 (0)	0 / 1 (0)	NA	NA	0 / 1 (0)	*0 / 1 (0)*	0 / 53 (0)	0 / 1 (0)	*0 / 1 (0)*
PTEN	9 / 54 (17)	0 / 1 (0)	NA	NA	1 / 1 (100)	*1 / 1 (100)*	9 / 55 (16)	1 / 1 (100)	*1 / 1 (100)*
p53	3 / 54 (6)	0 / 1 (0)	NA	NA	NA	*NA*	3 / 55 (5)	NA	*NA*
p-mTOR	29 / 50 (58)	1 / 1 (100)	NA	NA	0 / 1 (0)	*0 / 1 (0)*	30 / 51 (59)	0 / 1 (0)	*0 / 1 (0)*

Ki67	0 / 53 (0)	1 / 1 (100)	NA	NA	0 / 1 (0)	*0 / 1 (0)*	1 / 54 (2)	0 / 1 (0)	*0 / 1 (0)*

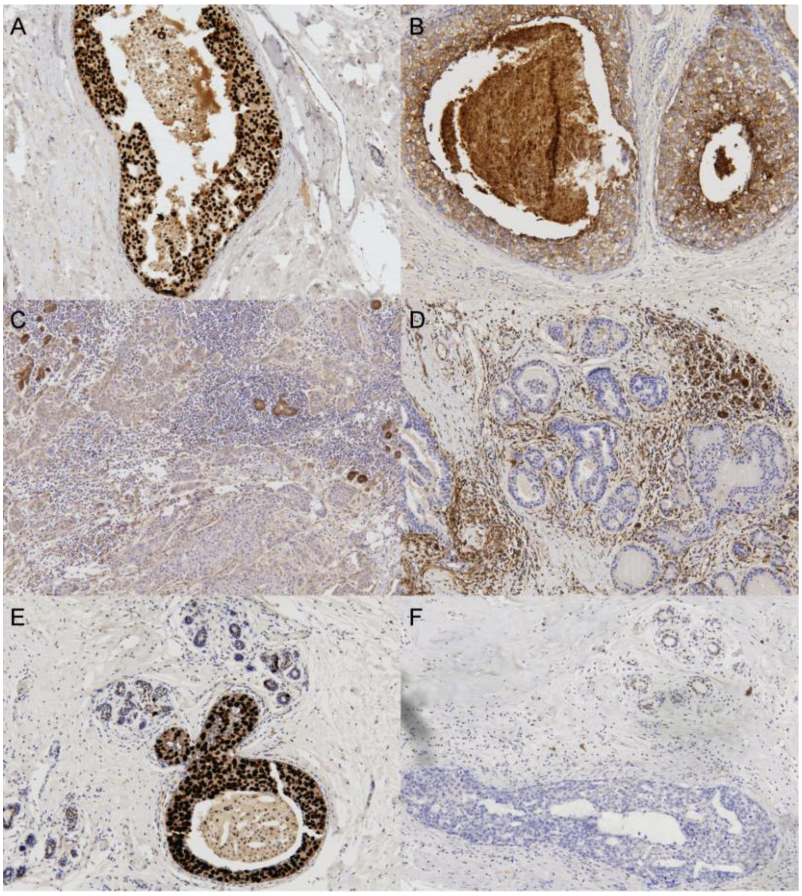

Supp Figure 1. Examples of biomarker differential expression in normal, DCIS and invasive carcinoma. Strong confluent nuclear expression of TOP2A is shown in A. EGFR membranous expression is shown in high grade DCIS with comedonecrosis (B). IGF1R expression tended to be retained in normal and benign lesions with loss occurring more frequently in in situ and invasive carcinoma. Scattered morphologically normal ducts are seen expressing IGF1R amongst non-expressing tumour (C). PTEN expression loss occurred in all lesion types especially when associated with invasive carcinoma. Retention of expression is seen in a lobular unit beside completely negative DCIS (D). Both p53 strong expression and complete absence of staining were more often present in DCIS associated with invasive carcinoma compared to pure examples (E, F). In both examples background ducts and lobules express predominantly weak p53.
